# CANGS: a user-friendly utility for processing and analyzing 454 GS-FLX data in biodiversity studies

**DOI:** 10.1186/1756-0500-3-3

**Published:** 2010-01-11

**Authors:** Ram Vinay Pandey, Viola Nolte, Christian Schlötterer

**Affiliations:** 1 Institut für Populationsgenetik, Veterinärmedizinische Universität Wien, Veterinärplatz 1, Vienna, Austria

## Abstract

**Background:**

Next generation sequencing (NGS) technologies have substantially increased the sequence output while the costs were dramatically reduced. In addition to the use in whole genome sequencing, the 454 GS-FLX platform is becoming a widely used tool for biodiversity surveys based on amplicon sequencing. In order to use NGS for biodiversity surveys, software tools are required, which perform quality control, trimming of the sequence reads, removal of PCR primers, and generation of input files for downstream analyses. A user-friendly software utility that carries out these steps is still lacking.

**Findings:**

We developed CANGS (**C**leaning and **A**nalyzing **N**ext **G**eneration **S**equences) a flexible and user-friendly integrated software utility: CANGS is designed for amplicon based biodiversity surveys using the 454 sequencing platform. CANGS filters low quality sequences, removes PCR primers, filters singletons, identifies barcodes, and generates input files for downstream analyses. The downstream analyses rely either on third party software (e.g.: rarefaction analyses) or CANGS-specific scripts. The latter include modules linking 454 sequences with the name of the closest taxonomic reference retrieved from the NCBI database and the sequence divergence between them. Our software can be easily adapted to handle sequencing projects with different amplicon sizes, primer sequences, and quality thresholds, which makes this software especially useful for non-bioinformaticians.

**Conclusion:**

CANGS performs PCR primer clipping, filtering of low quality sequences, links sequences to NCBI taxonomy and provides input files for common rarefaction analysis software programs. CANGS is written in Perl and runs on Mac OS X/Linux and is available at http://i122server.vu-wien.ac.at/pop/software.html

## Background

Next generation sequencing technologies have dramatically increased the sequence output at a substantially reduced cost. In addition to genome sequencing and transcriptome profiling, ultra-deep sequencing of short amplicons offers an enormous potential in clinical studies [1] and in studies of ecological diversity [2]. PCR amplicons of more than 400 bp can be sequenced in a massively parallel manner which allows building a fine-grained catalog of species abundance patterns in a broad range of habitats. This increase in the amount of sequence data requires efficient software tools for processing the raw data generated by next generation sequencers.

We developed CANGS - a flexible and user-friendly utility to trim sequences, filter low quality sequences, and produce input files for further downstream analyses. CANGS can be used to assign the taxonomic grouping based on similarity with sequences from the NCBI database [3].

CANGS has been developed for Mac OS X but it also works on Linux and any other Unix system. CANGS can be obtained from http://i122server.vu-wien.ac.at/pop/software.html. [See additional file 1 for the source code of CANGS, additional file 2 for test dataset of CANGS and additional file 3 for the CANGS user manual]

## Implementation

**Figure 1:**
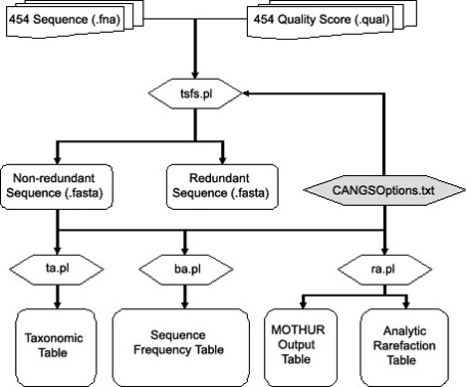
**The architecture of CANGS utility**. The four major components of the CANGS are tsfs.pl (Trimming Sequences and Filtering Sequences), ta.pl(Taxonomy Analysis), ba.pl (Blast Analysis) and ra.pl (Rarefaction Analysis). All these four components are connected to a single configuration file "CANGSOptions.txt" to take inputs.

CANGS software utility is written in PERL 5.8 using BioPerl [4]. The main workflow is depicted in Figure 1. The entire analysis is guided by a configuration file - CANGSOptions.txt and four PERL programs (tsfs.pl, ba.pl, ta.pl and ra.pl). CANGS can be run on Mac OS, Linux and other Unix like systems.

Required programs are BLAST [5] for the similarity search and MAFFT [6] for pairwise distance calculation. MOTHUR [7] and Analytic Rarefaction [8] are needed for estimation of the number of species (OTUs), and update_blastdb.pl [9] is required for downloading the BLAST database on a local computer.

## Results

### Schema for processing and analyzing 454 GS-FLX sequences

Figure 1 shows the way in which the CANGS utility processes 454-sequence data sets. The arrows illustrate the path of data flow. As a preparation step for CANGS, the options file *CANGSOptions.txt *needs to be customized. This file allows the user to specify all parameters needed for the processing of the 454 sequences. CANGS provides two layers of analysis: the Sequence Processing Layer is the first step, in which tsfs.pl trims the sequences (removal of PCR primers, adapter sequence and sample identifiers) and filters low quality sequences (sequences with Ns, singletons, and sequences with very low average quality score). The script tsfs.pl creates two high quality processed sequence data sets: 1) redundant sequences and 2) non-redundant sequences by using the user-defined parameters in the options file. The second step is the Sequence Analysis Layer in which three different programs are available to assign the newly sequenced reads to a taxonomic group (ta.pl), estimate the change in species composition among different samples (ba.pl), and to measure species richness (ra.pl).

### CANGS components

#### CANGS input customization

CANGS configuration file -- *CANGSOptions.txt*. CANGS was designed to allow a high flexibility for the user. In the options file the user defines the parameters that will be used by all CANGS modules. This simplified customization increases the usability and integration of the utility because the multiple programs can reference a single options file. The parameters include BLAST cutoff values, quality scores, PCR primers, barcodes, size range of PCR products etc.

#### Sequence trimming and quality filtering

The tsfs.pl (Trim Sequences and Filter low quality Sequences) program automates the processing of raw 454 sequences.

A typical 454 read consists of (5'- to 3'-end):

1. Sample Identifier (bar code)

2. Forward PCR Primer

3. Target Sequence

4. Reverse Primer

5. 454 adapter B

The goal of tsfs.pl is to obtain the high quality reads from pooled 454 sequences by trimming the raw sequences and filtering low quality reads which is done in seven steps.

##### 1. Removal of adapter B

based on the sequence of adapter B, as specified in the CANGSOptions.txt file, the 3'- end of each read is trimmed. It is possible to process only sequences with a perfect match to adapter B, but a pattern search that allows for imperfection in adapter B recovers more sequences.

##### 2. Filtering sequences with ambiguities

tsfs.pl removes reads with one or more Ns (unknown bases).

##### 3. Removal of singletons

to ameliorate the problem of sequencing errors tsfs.pl allows the user to remove very low frequency variants from the data set. Note that several data sets could be combined to minimize the removal of true low frequency sequence variants.

##### 4. Grouping of sequences according to bar codes

tsfs.pl distinguishes different samples based on the bar codes specified in the CANGSOptions.txt file and separates them into different data sets. This step is skipped when only a single sample is processed

##### 5. Filtering sequences according to length threshold

the tsfs.pl program removes sequence reads falling outside the size range specified in the options file.

##### 6. Removal of PCR primers

forward and reverse PCR primers are specified in the CANGSOptions.txt file and removed from the sequence. Only sequences with perfect identity to the specified PCR primers are processed. The 454 sequencing process preferentially generates length variants in homopolymers. As homopolymers can be as short as two bases and the target sequence is frequently not known, we developed a special procedure to recognize such sequencing errors at the end of the PCR primer:

for all sequences with the same PCR primers the tsfs.pl program scans 8 bp of the target sequence immediately adjacent to the PCR primer and identifies the most frequent 8 bp motif. Next, this consensus sequence is compared for the +1, and -1 offset of each sequence. For sequences with no 454 homopolymer mutation both the +1 and -1 offset results in many mismatches, but a read with a 454 homopolymer mutation at the end of the PCR primer will be very similar to either the +1 or -1 offset. We empirically determined that filtering reads with <3 mismatches very effectively removes reads with a 454 homopolymer mutation at the transition between target sequence and PCR primer. Hence, tsfs.pl removes all reads with <3 mismatches in the +1 or -1 offset.

##### 7. Quality filtering

CANGS averages the quality values for each base in a read. Quality values are taken from the .qual file after the values corresponding to adapter B, bar code and primer bases have been removed in step 1, step 4 and step 6, respectively. Sequence reads with a quality value lower than the threshold specified in the options file will be discarded. Note that the quality filtering may result in new singletons, which remain in the data set, as the quality filtering is the last step in the analysis.

After trimming the sequence reads, tsfs.pl creates a non-redundant sequence data set in order to reduce the computational burden for further analysis. In the non-redundant sequence data set each sequence variant is only represented once. Note that in this step indels are considered to be informative. Hence, two sequences differing only by an indel will be listed independently in the non-redundant data set. The frequency of each sequence in the non-redundant data set is included in the FASTA header. The output file contains non-redundant reads ranked based on copy number in descending order.

**Figure 2:**
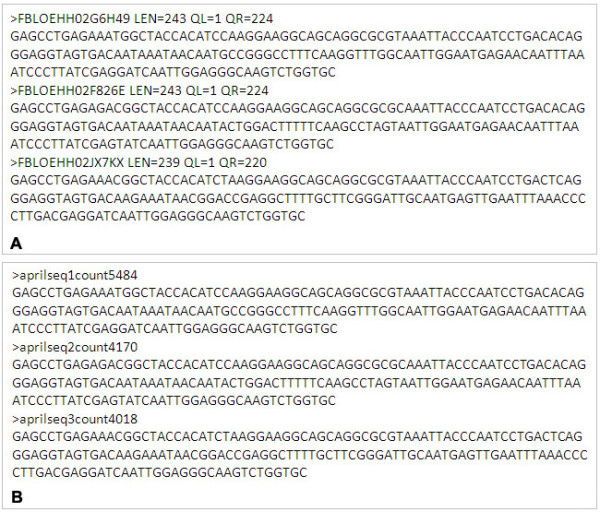
**An example output of the sequence trimming and quality filtering module in CANGS**. An example of the final output of Sequence trimming and quality filtering module (tsfs.pl). (A) example of redundant sequence data set and (B) example of the non-redundant sequence data set.

Figure 2B shows an example of a FASTA header for a non-redundant sequence.

To demonstrate the utility of CANGS, we used 454 sequences, which have been deposited in the NCBI database [NCBI: SRA008706.2]. This data set consists of 447,909 reads from the 18S rRNA gene obtained from 10 temporal freshwater samples. Applied to our example data set, the tsfs.pl program eliminated approximately 37% of all sequences (Table 1). Hence a total of **281,003 (~63%) **sequences could be used for downstream analyses. On Macintosh OS X version 10.6.2 with a single processor, CANGS (tsfs.pl) takes 6.5 hours for processing this data set. If the user skips the removal of singletons the tsfs.pl program takes only 20 minutes for the same data set.

**Table 1:** Number of reads eliminated at different steps of the tsfs.pl module

Order of steps	Steps	Total no. of sequences	No. of sequences considered	No. of sequences discarded
1	Removal of Adapter B	447,909	373,116	74,793

2	Filtering sequences with ambiguities	373,116	357,926	15,190

3	Removal of singletons	357,926	311,425	46,501

4	Grouping of sequences according to bar codes	311,425	306,042	5,383

5	Filtering sequences according to length threshold	306,042	305,884	158

6	Removal of PCR primers	305,884	282,053	23,831

7	Quality filtering	282,053	281,003	1,050

	Total Sequences	447,909	281,003	166,906

### Sequence Analysis

The sequence analysis module of CANGS performs various downstream analyses: 1) linking 454 reads with the taxonomic description of the most similar sequence from the NCBI database by ta.pl (Taxonomy Analysis) program, 2) measuring the overlap of OTUs between samples by ba.pl (Blast Analysis) program and 3) estimating species richness by the ra.pl (Rarefaction Analysis) module.

#### Taxonomy Analysis

**Figure 3:**
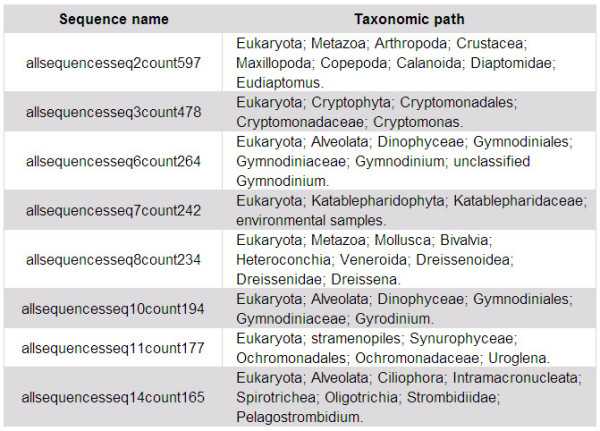
**An example output of taxonomy analysis in CANGS**. An example of final output of the Taxonomy Analysis (ta.pl) module. It is a tabular output, the columns from left to right are 1) Query sample non-redundant sequences 2) BLAST percent similarity 3) BLAST e-value 4) Closest NCBI sequence Accession ID 5) NCBI sequence species name and 6) Taxonomic path of the closest NCBI sequence.

ta.pl, this program classifies the processed 454 reads by assessing their similarity to taxonomic entries in the NCBI database. This analysis requires the nucleotide preformatted BLAST database from ftp://ftp.ncbi.nih.gov/blast/db/ to be installed, which is done using the perl program "*update_blastdb.pl" *[9]. The script ta.pl BLASTs the non-redundant sequences against this database. In a second step the best hit(s) from the BLAST search are used to retrieve the taxonomic path - either for all sequences or only for a taxonomic group of interest. In the case of multiple best hits with identical E-value, this program selects the hit with the most detailed taxonomic classification and links it with the non-redundant query sequence. If CANGS identifies a conflict we provide the option to assign taxonomic status by the majority rule. The partial output of this program is shown in Figure 3.

#### BLAST Analysis

**Figure 4:**
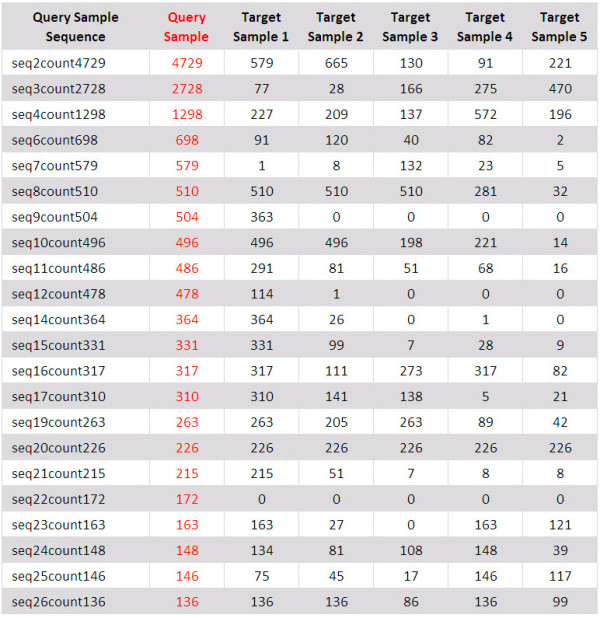
**An example output of BLAST analysis in CANGS**. An example of final output of the BLAST Analysis (ba.pl) module. It is a tabular output; the columns from left to right are 1) query sample non-redundant sequences 2) copy number of a query sequence in the same sample 3-7) copy number of query sequence in different target samples.

ba.pl. Studies of species diversity are frequently designed to compare species richness and species composition among different samples. The ba.pl (Blast Analysis) program performs a BLAST analysis of non-redundant sequences in one sample against non-redundant sequences in any number of other samples. For user convenience the ba.pl software automatically generates the BLAST database(s) required for the analyses. The output of the BLAST search(es), is parsed and a tabular output is created. As it may be of interest to group sequences with different similarities, the ba.pl program could be customized to group sequences up to a specified similarity. A similarity cutoff of 100 should be used to group only identical sequences (ignoring gaps). In the tabular output, the number of sequences shared between the two data sets is reported for every species as shown in Figure 4. The similarities given in the output are calculated as follows:

Hence, gaps are not considered.

#### Rarefaction Analysis

ra.pl. Several software packages exist for performing rarefaction analysis [7,8,10]. The script ra.pl (Rarefaction Analysis) program links the data processed by CANGS with two popular rarefaction analysis software packages with minimal user interference: MOTHUR [7] and Analytic Rarefaction [8]. For MOTHUR ra.pl is calculating the pairwise genetic distance by using the *"mafft-distance" *program of MAFFT executables [6]. The *mafft-distance *program takes non-redundant sequences generated by tsfs.pl as input and gives the corresponding genetic distance table as output. For the Analytic Rarefaction software, ra.pl first calculates the abundance of each sequence in the data set using BLAST, as described above. Compared to pattern matching this procedure allows to consider sequences with gaps jointly.

## Conclusion

CANGS is a user-friendly tool for primer clipping and quality filtering of 454 sequences. CANGS is primarily designed to handle data from amplicon resequencing projects in the context of diversity studies. The tool can be downloaded at http://i122server.vu-wien.ac.at/pop/software.html.

## Availability & requirements

**Project name**: CANGS--Cleaning and Analyzing 454 GS-FLX sequences.

**Availability**: http://i122server.vu-wien.ac.at/pop/software.html

**Operating System**: Mac OS X, Linux and any other UNIX like system

**Programming language**: Perl 5.8.8

**Other requirements**: BioPerl, BLAST, MAFFT, MOTHUR, Analytic Rarefaction.

**License**: GNU General Public License.

**Any restrictions to use by non-academics**: license needed.

## Competing interests

The authors declare that they have no competing interests.

## Authors' contributions

VN and CS designed the study. RVP analyzed and wrote the code. RVP wrote the draft of the manuscript and VN, CS and RVP revised it. All authors read and approved the final manuscript.

## Supplementary Material

Additional file 1 **CANGS source code**. This file contains source code of CANGS utility and CANGS configuration file. Click here for file

Additional file 2 **Input test data set for CANGS**. This file contains 454 GS-FLX reads in FASTA file format and quality score file as sample input data set to run all modules of the CANGS utility. Click here for file

Additional file 3 **CANGS User Manual**. A user manual in PDF file format; it describes how to set working environments of this software and how to use the modules of the CANGS utility. Click here for file
