# Supplementary material for: CANGS: a user-friendly utility for processing and analyzing 454 GS-FLX data in biodiversity studies
Source: BMC Res Notes. 2010 Jan 11;3:3. doi: 10.1186/1756-0500-3-3 (PMC2830946; doi:10.1186/1756-0500-3-3)
Supplement: Additional file 3 — CANGS User Manual. A user manual in PDF file format; it describes how to set working environments of this software and how to use the modules of the CANGS utility. [file 1756-0500-3-3-S3.PDF]

CANGS Manual – version 1.0

Ram Vinay Pandey, Viola Nolte, Christian Schlötterer

September 08, 2009

Table of contents

1. Background …………………………………………………………………... 2

2. Obtaining the CANGS ………...…………………………………………….... 2

    2.1 Getting Perl …………………………………………………………… 3

    2.2 Installing Bioperl …………………………………………………... 3

    2.3 Getting stand-alone blast ……………………………………… 4

    2.4 Installing MAFFT program ……………………………………….. 4

    2.5 Installing MOTHUR program …………………………………….. 5

    2.6 Getting Analytic Rarefaction program …………………………... 5

    2.7 Downloading pre-formatted NCBI BLAST database…………... 6

    2.8 Download test data set …………………………………………… 6

3. Using CANGS (short version) ……………………………………….…... 7

4. Using CANGS (extended version)………………………………………….. 8

5. Processing layer program ..………………………………………………..... 9

    5.1 Process sequence program (tsfs.pl) ...…………..…………….....… 9

6. Analysis Layer Program ……………………………………………........ 14

    6.1 ta.pl (Taxonomy Analysis) program …………………………….. 14

    6.2 ba.pl (Blast Analysis) program ………………………………...... 21

    6.2 ra.pl (Rarefaction Analysis) program ……………………………. 25

1. BACKGROUND:

CANGS is a utility, which is designed to automate the process of trimming sequences, filtering low quality sequences and performing various analyses for diversity study. There are basically two layers in CANGS 1) processing layer and 2) Analysis Layer.

1.1 Processing Layer:

tsfs.pl -- (Trim Sequences and Filter Low Quality Sequences), this program trims the raw sequences: it removes PCR primers, adapter sequence and sample identifiers) and filters the low quality sequences.

The outputs are high quality sequences that can be used for further analysis.

1.2 Analysis Layer:

ta.pl -- (Taxonomy Analysis), this program provides all taxonomic information for the 454 GS FLX sequences as available in NCBI to explore the taxonomic group of interest.

ba.pl -- (Blast Analysis), if the user wants to compare multiple samples, this program produces a sequence frequency table, which shows how much overlap in species composition exists between different samples. This will give an idea of the species turnover and fluctuations in diversity.

ra.pl -- (Rarefaction Analysis), by incorporating two independent packages for performing rarefaction analysis, this program produces estimates of the number of species from a given number of sequences.

2. OBTAINING THE CANGS PACKAGE:

The CANGS package is located at http://i122server.vu-wien.ac.at/pop/software.html

The package has been developed using the programs below. If any of these programs are not currently available locally, they will need to be downloaded and installed in the directory path or in their proper places.

1. Perl, version 5.8.8 or later (http://www.perl.org/)
2. Bioperl, version 1.4 (http://www.bioperl.org)
3. Standalone Blast, version 2.2.21 (ftp://ftp.ncbi.nih.gov/blast/executables/LATEST)
4. MAFFT, version 6.716 (http://align.bmr.kyushu-u.ac.jp/mafft/software/source.html)
5. MOTHUR, version 1.7.0 (http://schloss.micro.umass.edu/mothur/Mothur_v.1.7.0)
6. Analytic Rarefaction, version 1.4 (http://www.uga.edu/~strata/software/)
7. NCBI web BLAST (http://blast.ncbi.nlm.nih.gov/Blast.cgi)
8. NCBI (http://www.ncbi.nlm.nih.gov/)

2.1 Getting Perl:

Perl is inbuilt in all Unix systems.

2.2 Installing Bioperl:

You can now follow the Unix instructions for installing Bioperl manually:

1) Download the "bioperl-1.4.tar.gz" version of the bioperl package from http://bioperl.org/DIST/bioperl-1.4.tar.gz
2) Open a terminal window and change to the directory where the tar file was downloaded.

   $>cd /Users/username/Downloads/

3) Extract the archive in the normal way, by using the following command.

   $>tar –vzvf bioperl-1.4.tar.gz

4) Change to the newly created directory "bioperl-1.4"

   $>cd bioperl-1.4

5) Type 'perl Makefile.PL' and answer the questions appropriately.

   $>perl Makefile.PL

6) Type 'sudo make' to make all configuration files.

   $>sudo make

7) Type 'sudo make test'. All the tests should pass, but if they don't then also your usage of BioPerl may not be affected by the failure, so you can choose to continue anyway.

$>sudo make test

8) Type 'sudo make install' to install BioPerl.

$>sudo make install

Note: sudo is used to install as root or system administrator.

2.3 Getting Standalone Blast:

1) Download Latest BLAST from ftp://ftp.ncbi.nih.gov/blast/executables/LATEST

2) Open a terminal window and change to the directory where the tar file was downloaded.

$>cd /Users/username/Downloads/

3) Open a terminal window and enter to extract the archive:

$> tar –xzvf blast-x.x.xx.tar.gz

4) Put this BLAST"s directory/folder full path to the option BLAST_DIRECTORY in CANGSOptions.txt.

2.4 Installing MAFFT Program:

COMPILE & INSTALL MAFFT

1) Download the latest MAFFT release from http://align.bmr.kyushu-u.ac.jp/mafft/software/source.html.
2) Open a terminal window and change to the directory where the tar file was downloaded.

$>cd /Users/username/Downloads/

3) Extract the archive in the normal way explain as above.

$>*tar -xzvf mafft-6.708-without-extensions-src.tgz*

4) Change to the newly created directory "mafft-6.708-without-extensions"

$>*cd mafft-6.708-without-extensions*

5) Run these commands as root user (System Administrator)
$> cd core
$> sudo make clean
$> sudo make
$> sudo make install

2.5 Installing MOTHUR on UNIX:

Please follow the instructions below which are taken from the MOTHUR web page (http://www.mothur.org/wiki/Main_Page):

"In the Mac OSX and Linux-type environments, you need to have a C++ compiler installed. This is typically installed with most linux-type operating systems and can be found on the Mac OSX installation CD/DVD. For Mac OSX users, you need to install the Xcode developer's tools, which also can be found on the Mac OSX installation DVD. After downloading mothur, decompress it".

Open a terminal window and change to the directory where the zip file was downloaded.

$>cd /Users/username/Downloads/

$> unzip mothur.zip

This will generate a mothur-1.7.1 folder. Now move into the mothur folder and compile mothur:

$> cd mothur-1.7.1

$> sudo make

2.6 Getting Analytic Rarefaction:

5) Download the latest Analytic Rarefaction from http://www.uga.edu/~strata/software/zip/AnalyticRarefaction.zip.

6) Open a terminal window and enter to extract the archive:

$> unzip AnalyticRarefaction.*zip*

7) You will get the application file “Analytic Rarefaction .app”

8) Move this file in “ra-output/outputAnalyticRarefaction” subdirectory/subfolder.

2.7 Downloading pre-formatted NCBI BLAST database:

The pre-formatted non-redundant NCBI nucleotide BLAST is required only for ta.pl (Taxonomy Analysis) module of CANGS. Before running the ta.pl program user has to run the “***update_ncbi_blastdb.pl***” program available with CANGS. The following command should be used to download nr BLAST database. The program update_ncbi_blastdb.pl downloads and uncompressed NCBI preformatted BLAST databases in the nr-blastdb sub directory/sub folder. Finally put the full path of this sub directory/sub folder to the option NCBI_BLAST_DB_DIRECTORY in CANGSOptions.txt file.

“The update_ncbi_blastdb.pl is a modified version of original update_blastdb_pl [http://www.ncbi.nlm.nih.gov/BLAST/docs/update_blastdb.pl]. This modification was done according to CANGS’s ta.pl program requirement. Therefore user is advised to use modified program (update_ncbi_blastdb.pl), which is available with CANGS1.0 source code”.

CANGS1.0$>perl update_ncbi_blastdb.pl nt

2.8 Download test data set:

The test data set can be obtained from http://i122server.vu-wien.ac.at/pop/software.html. The default input options given in CANGSOptions.txt file can be used with this data set.

All testing was done on a Macintosh OS X 10.5.8 system and should work on any Unix system.

3. Using CANGS (short version):

1. Create subdirectories/subfolders ‘tsfs-input’, ‘ba-input’, ‘ta-input’ and ‘ra-input’ if not already created in any location of server or computer. These subdirectories/subfolders name is case sensitive (give name in lower case).

2. Put input FASTA files in these input directories for corresponding program.

3. Customize options file (CANGSOptions.txt).

4. Run tasfs.pl

5. Run ta.pl or ba.pl or ra.pl

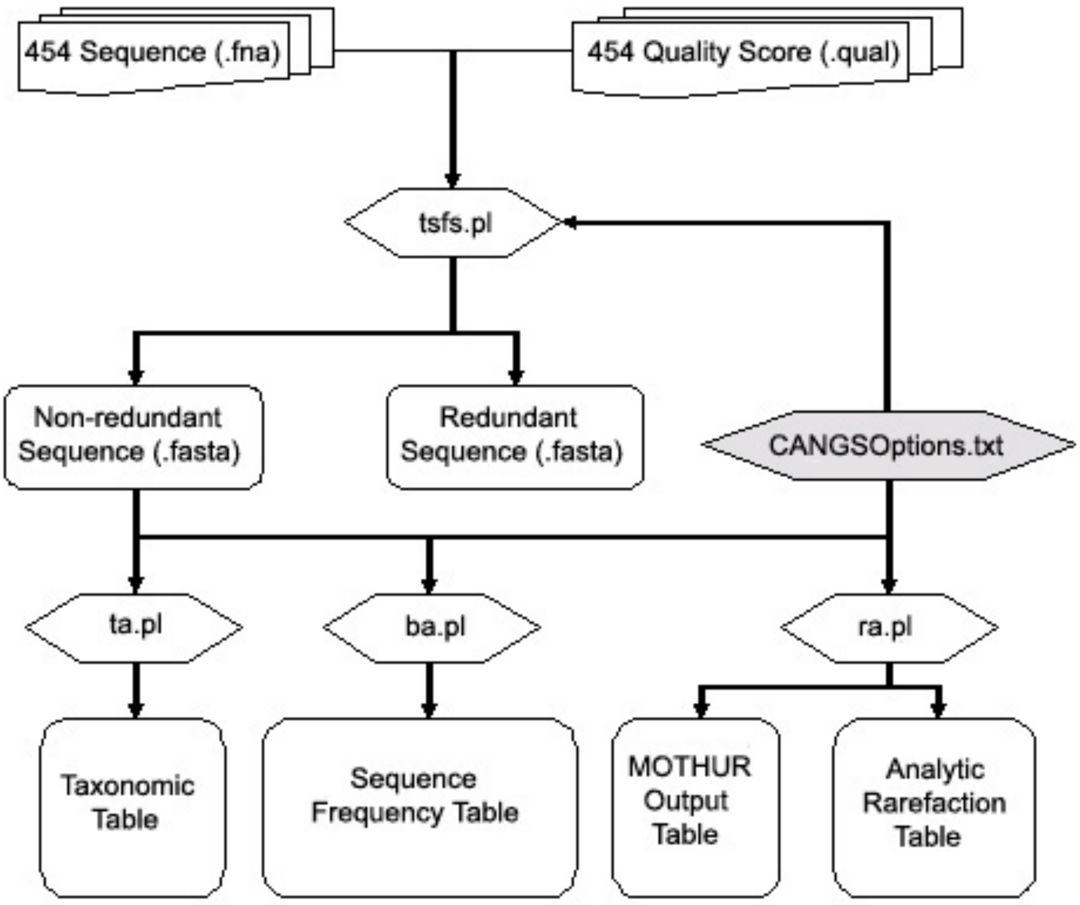

Figure 1 the architecture of CANGS utility

4. Using CANGS (extended version):

To use CANGS, one must first create 4 subdirectories/subfolders *tsfs-input*, *ba-input*, *ta-input* and *ra-input* for *tsfs.pl*, *ba.pl*, *ta.pl* and *ra.pl* respectively anywhere in server or computer. These subdirectories/subfolders name is case sensitive (give name in lower case). Therefore it should be always same.

In tsfs-input subdirectory/subfolder 454 sequence (.fasta OR .fna OR .fa) and 454 quality score (.qual), two FASTA files should be placed -- one containing the 454 sequences and one containing the 454 quality scores.

In ta-input subdirectory/subfolder put “non-redundant sequence fasta” files (created by tsfs.pl program). Before running the ta.pl (Taxonomy Analysis) module download preformatted NCBI BLAST database as mentioned in section 2.7.

In ba-input subdirectory/subfolder put “non-redundant sequence fasta” files (created by tsfs.pl program).

In ra-input subdirectory/subfolder put “non-redundant sequence/redundant fasta” files (created by tsfs.pl program).

5. Processing Layer Program:

5.1 tsfs.pl -- (Trim sequences and filter low quality sequences) program:

This program processes raw 454 sequences (amplicons) in the following way:

Trimming Sequences:

1) Trims adapter B sequence from 3' end (option: "ADAPTER_B_SEQUENCE" in the CANGSOptions.txt file)
2) Trims the sample identifier tag (options: "SAMPLE_COUNT", "SAMPLE_TAG_LENGTH" and section "barcode" as shown in Figure 3B)
3) Trims forward and reverse primer sequences (options: "UPPER_SEQUENCE_LENGTH_CUTOFF_FOR_PRIMER_CLIPPING", "LOWER_SEQUENCE_LENGTH_CUTOFF_FOR_PRIMER_CLIPPING", "SEQUENCE_LENGTH_TO_FIND_HOMOPOLYMER_MUTATION", "FORWARD_PRIMER", "REVERSE_PRIMER",)

Filtering Low Quality Sequences:

1) Filters sequences in which Adapter B sequence was not found.
2) Filters sequences with Ns.
3) Filters sequences, which are single copy in the sequence set (singletons) (option: "SEQUENCE_COPY_NUMBER").

As a result, there are at least 2 copies of each sequence present in the sequence data set before trimming primer sequences

4) Before primer clipping this program checks for sequencing artifacts, which add or delete nucleotides during the 454 sequencing process. Reads for which an indel is identified at the transition to the sequencing primer are also removed.
5) Primers are clipped.
6) After primer clipping sequences are filtered based on a given average quality score. In this filtering one may loose one or many copies of sequences. Therefore, despite that singletons were filtered out in step 3, the sequence data set will contain singletons again after running tsfs.pl (option: "AVERAGE_CUTOFF_QUALITY_VALUE").

Note:

1. Adapter-B trimming could be skipped by leaving ADAPTER_B_SEQUENCE option blank in CANGSOptions.txt.
2. Singltons removal could be skipped by setting 0 to the option SEQUENCE_COPY_NUMBER in CANGSOptions.txt.
3. Length wise sequence filtering could be skipped by leaving the options UPPER_SEQUENCE_LENGTH_CUTOFF_FOR_PRIMER_CLIPPING and

LOWER_SEQUENCE_LENGTH_CUTOFF_FOR_PRIMER_CLIPPING blank in CANGSOptions.txt.

4. Multiple Forward and reverse primers could be trimmed by tsfs.pl program by putting primer sequences with options FORWARD_PRIMER and REVERSE_PRIMER in CANGSOptions.txt file.
5. Primer trimming could be skipped by leaving options FORWARD_PRIMER and REVERSE_PRIMER blank in CANGSOptions.txt file.

6. Sequences with low average quality score filtering could be skipped by leaving the option AVERAGE_CUTOFF_QUALITY_VALUE blank in CANGSOptions.txt file.

Process Sequence program (tsfs.pl) Input:

1) Fasta file containing 454 Raw Sequence:

>E9LAHD006DM038 LEN=241 QL=1 QR=222
TAGG ATTAGGGTTCGATTCCGGAGAGG
GAGCCTGAGAAACGGCTACCACATCTAAGGAAGGCAGCAGGCGCGCAAATTACCCAAT
CCTGACGCAGGGAGGTAGTGACAAGAAATAACAATACAGGGCATATCTGTCTTGTAATT
GGAATGAGTAAACTTTAAATCACTTTACGAGTATCAATTGGAGGGCAAGTCTGGTGC
CAGCACCCGCGGTAATTCCAG CTGAGCGGGCTGGCAAGGC

1. Sample Identifier
2. Forward Primer
3. Real Sequence
4. Reverse Primer
5. Adapter B

2) Fasta file containing 454 Raw Sequence Quality Score:

>E9LAHD006DM038 LEN=241 QL=1 QR=222
37 37 37 37 37 37 37 37 37 37 37 37 37 37 37 37 37 37 40 40 40 40 40 40 40 38 38 38 40 40
40 40 40 40 40 40 40 40 40 40 40 40 40 40 37 37 37 37 37 37 37 37 37 37 37 37 37 37 37 37
37 37 37 37 37 37 37 37 37 37 37 37 37 37 37 37 37 37 37 37 37 37 37 37 37 37 37 37 37 37
37 37 37 37 37 37 37 37 37 37 37 37 37 37 37 37 37 37 37 37 37 37 37 37 37 37 37 37 37 37
37 37 37 37 37 37 37 37 37 37 37 37 37 37 37 37 37 37 37 37 37 37 37 37 37 37 37 37 37 37
37 37 37 38 34 34 34 37 37 37 37 37 37 37 37 37 37 37 37 37 37 37 37 37 37 37 37 37 37 37
37 37 37 37 37 37 37 37 37 37 37 37 37 37 37 37 37 37 37 37 37 37 37 37 37 37 37 37 37 37
37 37 37 37 37 37 40 40 40 40 40 40 40 40 40 40 40 40 40 38 38 38 40 40 40 40 38 38 38 38
40

Process Sequence program (tsfs.pl) Output:

```
>FBLOEHH02G6H49 LEN=243 QL=1 QR=224
GAGCCTGAGAAATGGCTACCACATCCAAGGAAGGCAGCAGGCGCGTAAATTACCCAATCCTGACACAG
GGAGGTAGTGACAATAAATAACAATGCCGGGCCTTTCAAGGTTTGGCAATTGGAATGAGAACAATTTAA
ATCCCTTATCGAGGATCAATTGGAGGGCAAGTCTGGTGC
>FBLOEHH02F826E LEN=243 QL=1 QR=224
GAGCCTGAGAGACGGCTACCACATCCAAGGAAGGCAGCAGGCGCGCAAATTACCCAATCCTGACACAG
GGAGGTAGTGACAATAAATAACAATACTGGACTTTTTCAAGCCTAGTAATTGGAATGAGAACAATTTAA
ATCCCTTATCGAGTATCAATTGGAGGGCAAGTCTGGTGC
>FBLOEHH02JX7KX LEN=239 QL=1 QR=220
GAGCCTGAGAAACGGCTACCACATCTAAGGAAGGCAGCAGGCGCGTAAATTACCCAATCCTGACTCAG
GGAGGTAGTGACAAGAAATAACGGACCGAGGCTTTTGCTTCGGGATTGCAATGAGTTGAATTTAAACCC
CTTGACGAGGATCAATTGGAGGGCAAGTCTGGTGC
```

A

```
>aprilseq1count5484
GAGCCTGAGAAATGGCTACCACATCCAAGGAAGGCAGCAGGCGCGTAAATTACCCAATCCTGACACAG
GGAGGTAGTGACAATAAATAACAATGCCGGGCCTTTCAAGGTTTGGCAATTGGAATGAGAACAATTTAA
ATCCCTTATCGAGGATCAATTGGAGGGCAAGTCTGGTGC
>aprilseq2count4170
GAGCCTGAGAGACGGCTACCACATCCAAGGAAGGCAGCAGGCGCGCAAATTACCCAATCCTGACACAG
GGAGGTAGTGACAATAAATAACAATACTGGACTTTTTCAAGCCTAGTAATTGGAATGAGAACAATTTAA
ATCCCTTATCGAGTATCAATTGGAGGGCAAGTCTGGTGC
>aprilseq3count4018
GAGCCTGAGAAACGGCTACCACATCTAAGGAAGGCAGCAGGCGCGTAAATTACCCAATCCTGACTCAG
GGAGGTAGTGACAAGAAATAACGGACCGAGGCTTTTGCTTCGGGATTGCAATGAGTTGAATTTAAACCC
CTTGACGAGGATCAATTGGAGGGCAAGTCTGGTGC
```

B

Figure 2: shows output of tsfs.pl program A) Redundant Sequences and B) Non-redundant Sequences

How to run the Processing Sequence (tsfs.pl) program?

Customize the following input options for tsfs.pl program in "*CANGSOptions.txt*" file and follow the given steps below:

```
#########################################################################################
## tsfs.pl options
#########################################################################################

# 454 RUN identifier (it will be helpful to organize the outout for multiple runs)
# In case of multiple runs analysis.
454_RUN_IDENTIFIER  mondsee

# Adapter B sequence in 5' to 3' direction
# If there is no Adapter B, then leave this field blank
ADAPTER_B_SEQUENCE  GCCTTGCCAGCCCGCTCAG

# Sequences to be removed with minimum copy number in whole data set: 1
# To skip the removal of least frequent sequences put 0
SEQUENCE_COPY_NUMBER  1

# Number of samples present in whole sequence deta set: 10
# If the whole data set contains single sample then put 1
SAMPLE_COUNT    10

#Sample identifier tag length: 4
SAMPLE_TAG_LENGTH   4

#Maximumread read length before primers clipping: 1000
# If there is no upper length limit, then leave this field blank
UPPER_SEQUENCE_LENGTH_CUTOFF_FOR_PRIMER_CLIPPING    1000

#Minimum read length before primers clipping: 200
# If there is no lower length limit, then leave this field blank
LOWER_SEQUENCE_LENGTH_CUTOFF_FOR_PRIMER_CLIPPING    200

#Sequence length adjecent to forward and reverse primers to check homopolymer mutation: 8
SEQUENCE_LENGTH_TO_FIND_HOMOPOLYMER_MUTATION    8

#Forward primers sequence: ATTAGGGTTCGATTCCGGAGAGG
# If there is no Forward primer(s), then leave this field blank
FORWARD_PRIMER  ATTAGGGTTCGATTCCGGAGAGG
FORWARD_PRIMER
FORWARD_PRIMER

#Reverse primers sequence in 5' to 3' direction: CTGGAATTACCGCGGSTGCTG
# If there is no Reverse primer(s), then leave this field blank
REVERSE_PRIMER  CTGGAATTACCGCGGSTGCTG
REVERSE_PRIMER
REVERSE_PRIMER


#Minumum average quality score: 25
# If there is no Quality score filtering, then leave this field blank
AVERAGE_CUTOFF_QUALITY_VALUE  25


# barcode    Sample Name Sample Identifier tag
BARCODE Sample1 AACG
BARCODE Sample2 TAGG
BARCODE Sample3 ACAG
BARCODE Sample4 AGCG
BARCODE Sample5 CTAG
BARCODE Sample6 ACGG
BARCODE Sample7 ATCG
BARCODE Sample8 ACTG
BARCODE Sample9 ATAG
BARCODE Sample10    ATGG

#tsfs.pl output sub directory
TSFS_OUTPUT_DIRECTORY   tsfs-output
```

Figure 3: shows how to customize the input options for tsfs.pl program

Step1: In "tsfs-input" subdirectory/subfolder the two FASTA files "454 sequence" and "454 quality score" should be placed -- one containing the 454 sequences (with file extension .fna | .fasta | .fa) and one containing the 454 quality scores (with file extension .qual).

Note: The name of sequence and quality value files should be same

Step2: Run the Program "tsfs.pl" as shown in figure 4. In this, give the sequence and quality value files as input with full path in the terminal window (drag 454 sequence and 454 quality score file on command after typing "perl tsfs.pl")

For Example:

$>perl tsfs.pl CANGSOptions.txt tsfs-input/test-dataset.fna tsfs-input/test-dataset.qual

```
i122mc100:CANGS1.1 ramvinay$ perl tsfs.pl CANGSOptions.txt tsfs-input/test-dataset.fna tsfs-input/test-dataset.qual

        ****** Adapter B trimming OPTIONS ******

        1. WITH PERFECT SEARCHING

        2. WITH PATTERN SEARCHING

        Your Choice: 2
```

Figure 4: shows how to run the process sequence program — tsfs.pl (by giving sequence and quality value files on command line)

6. Analysis Layer Program:

All programs (ta.pl, ba.pl and ra.pl) take non-redundant sequences as input, which will be generated by the tsfs.pl program and located in “tsfs-output/nonRedundant-SampleWise-Sequence”.

6.1 ta.pl -- (Taxonomy Analysis) program: This program gives all possible taxonomic information for the NGS sequences to explore the taxonomic group of interest
This program works in following way:

1) It BLASTs all sequences against the NCBI nucleotide blast database (option: "BLAST_DIRECTORY" and “NCBI_BLAST_DB_DIRECTORY” in CANGSOptions.txt file).
2) It parses the BLAST output to obtain the accession IDs of closely related NCBI sequences.
3) It retrieves the GenBank format file for all Accession IDs (accession IDs found in the BLAST parsing output) from NCBI.
4) Parses GenBank files to pick the Taxonomic information.
5) Assigns the taxonomic group to the newly sequenced reads (option: " MEJORITY_PERCENTAGE")

Blast output-parsing criteria:

All BLAST hits, with e-value equal to the e-value of the best hit and % similarity above 90, are considered.

Taxonomy Analysis program (ta.pl) Output:

Sequence name	Taxonomic path
allsequencesseq2count597	Eukaryota; Metazoa; Arthropoda; Crustacea; Maxillopoda; Copepoda; Calanoida; Diaptomidae; Eudiaptomus.
allsequencesseq3count478	Eukaryota; Cryptophyta; Cryptomonadales; Cryptomonadaceae; Cryptomonas.
allsequencesseq6count264	Eukaryota; Alveolata; Dinophyceae; Gymnodiniales; Gymnodiniaceae; Gymnodinium; unclassified Gymnodinium.
allsequencesseq7count242	Eukaryota; Katablepharidophyta; Katablepharidaceae; environmental samples.
allsequencesseq8count234	Eukaryota; Metazoa; Mollusca; Bivalvia; Heteroconchia; Veneroida; Dreissenoidea; Dreissenidae; Dreissena.
allsequencesseq10count194	Eukaryota; Alveolata; Dinophyceae; Gymnodiniales; Gymnodiniaceae; Gyrodinium.
allsequencesseq11count177	Eukaryota; stramenopiles; Synurophyceae; Ochromonadales; Ochromonadaceae; Uroglena.
allsequencesseq14count165	Eukaryota; Alveolata; Ciliophora; Intramacronucleata; Spirotrichea; Oligotrichia; Strombidiidae; Pelagostrombidium.

Figure 5: shows the partial output of the ta.pl program

How to run the Taxonomy Analysis (ta.pl) program?

Customize the following input options for ta.pl program in "*CANGSOptions.txt"* file and follow the given steps below:

```
##########################################################################################
## ta.pl options
##########################################################################################

# ta.pl input sequences sub directory
TA_INPUT_DIRECTORY  ta-input

# ta.pl output sub directory
TA_OUTPUT_DIRECTORY   ta-output

# The location where you will put pre-formatted NCBI BLAST database
NCBI_BLAST_DB_DIRECTORY   nr-blastdb

# The executable BLAST location
# Enter the full path to the directory containing the BLAST executables
BLAST_DIRECTORY blast/bin

# The percentage to decide the mejority for assigning the taxonomic path
MEJORITY_PERCENTAGE  66
```

Figure 6: shows how to customize the input options for ta.pl program

Note: The Taxonomic Keywords should be as per NCBI Taxonomy database information.

Step1: Put all non-redundant FASTA sequence files in "ta-input" subdirectory/subfolder.

Step2: Run the Program "ta.pl" as shown in figure 7 with choice 1 this program will run the BLAST search, parse the BLAST output files and list all Accession IDs in "*allGBKaccessionIDs.txt" file.*

```
i122mc100:CANGS1.1 ramvinay$ perl ta.pl CANGSOptions.txt

        ****** TAXONOMIC ANALYSIS PROGRAM MENU ******

        1. BLAST SEARCH [1]

        2. BLAST OUTPUT PARSING [2]

        3. PARSING GEN BANK FILES & ASSIGNING THE TAXONOMIC PATH TO 454 SEQUENCES [3]

        4. Press [RETURN] to exit the program

        Your Choice: 1
```

Figure 7: shows how to run the program for BLAST search

Step3: Run the Program “ta.pl” as shown in figure 8 with choice 2, this program parses the BLAST search output from step2 to get all best hits and lists all Accession IDs in *“allGBKaccessionIDs.txt” file.*

```
i122mc100:CANGS1.1 ramvinay$ perl ta.pl CANGSOptions.txt

        ****** TAXONOMIC ANALYSIS PROGRAM MENU ******

        1. BLAST SEARCH [1]

        2. BLAST OUTPUT PARSING [2]

        3. PARSING GEN BANK FILES & ASSIGNING THE TAXONOMIC PATH TO 454 SEQUENCES [3]

        4. Press [RETURN] to exit the program

        Your Choice: 2
```

Figure 8: shows how to run the program for BLAST parsing

Step4: Take the output file of Step 3 from **“**ta-output/blastParsingOutput” subdirectory/subfolder” named as “*allGBKaccessionIDs.txt”.* This file contains comma separated GenBank accession IDs of the closely related sequences. Select “Nucleotide” search and paste the accession IDs in search box. Download the GenBank record for all accession IDs from NCBI (http://www.ncbi.nlm.nih.gov/) as shown in Figure 9A, 9B & 9C.

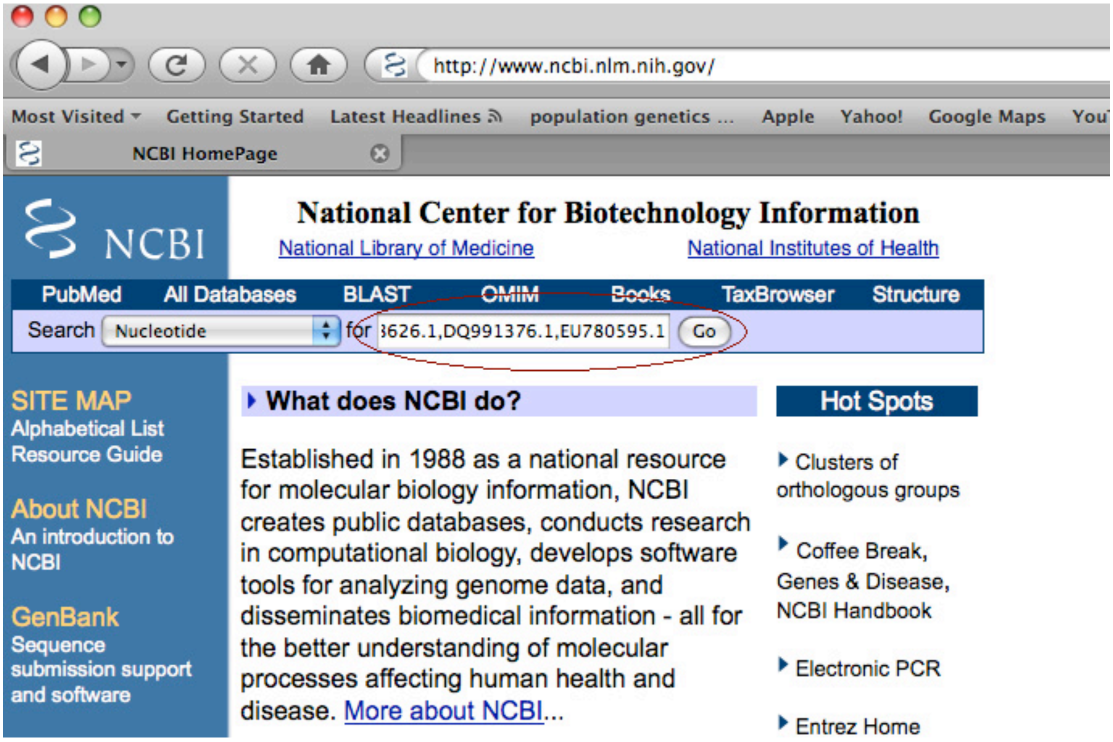

Figure 9A: shows how to search the GenBank record for all Accession IDs

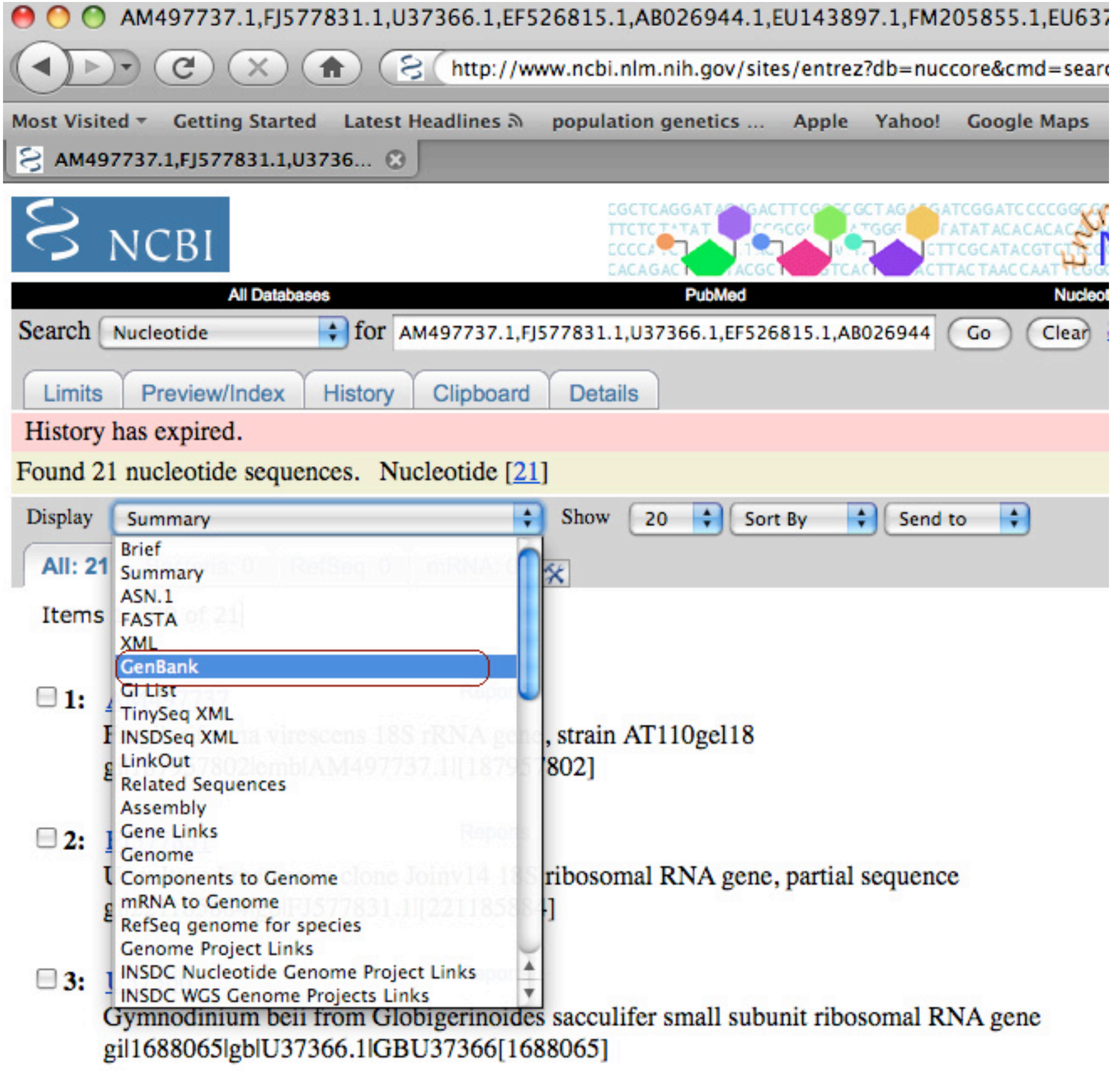

Figure 9B: shows how to get the GenBank full record for all accession IDs

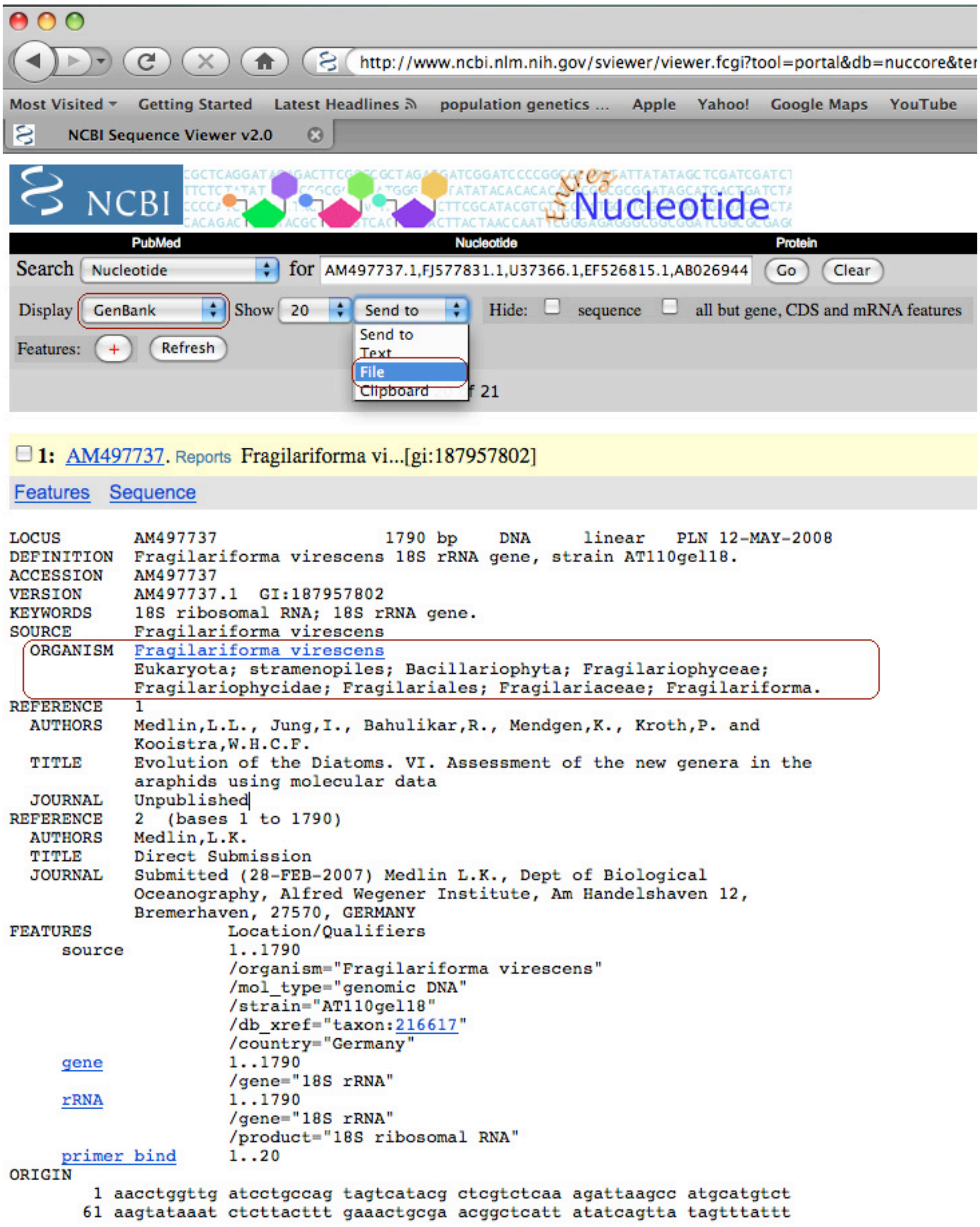
http://www.ncbi.nlm.nih.gov/sviewer/viewer.fcgi?tool=portal&db=nuccore&ter
Most Visited
Getting Started
Latest Headlines
population genetics ...
Apple
Yahoo!
Google Maps
YouTube
NCBI Sequence Viewer v2.0
NCBI
Entrez Nucleotide
PubMed
Nucleotide
Protein
Search
Nucleotide
for
AM497737.1,FJ577831.1,U37366.1,EF526815.1,AB026944
Go
Clear
Display
GenBank
Show
20
Send to
Hide:
sequence
all but gene, CDS and mRNA features
Features:
Refresh
Send to
Text
File
Clipboard
f 21
1: AM497737. Reports Fragilariforma vi...[gi:187957802]
Features
Sequence
LOCUS AM497737 1790 bp DNA linear PLN 12-MAY-2008
DEFINITION Fragilariforma virescens 18S rRNA gene, strain AT110gel18.
ACCESSION AM497737
VERSION AM497737.1 GI:187957802
KEYWORDS 18S ribosomal RNA; 18S rRNA gene.
SOURCE Fragilariforma virescens
ORGANISM Fragilariforma virescens
Eukaryota; stramenopiles; Bacillariophyta; Fragilariophyceae;
Fragilariophycidae; Fragilariales; Fragilariaceae; Fragilariforma.
REFERENCE 1
AUTHORS Medlin,L.L., Jung,I., Bahulikar,R., Mendgen,K., Kroth,P. and
Kooistra,W.H.C.F.
TITLE Evolution of the Diatoms. VI. Assessment of the new genera in the
araphids using molecular data
JOURNAL Unpublished
REFERENCE 2 (bases 1 to 1790)
AUTHORS Medlin,L.K.
TITLE Direct Submission
JOURNAL Submitted (28-FEB-2007) Medlin L.K., Dept of Biological
Oceanography, Alfred Wegener Institute, Am Handelshaven 12,
Bremerhaven, 27570, GERMANY
FEATURES Location/Qualifiers
source 1..1790
/organism="Fragilariforma virescens"
/mol_type="genomic DNA"
/strain="AT110gel18"
/db_xref="taxon:216617"
/country="Germany"
gene 1..1790
/gene="18S rRNA"
rRNA 1..1790
/gene="18S rRNA"
/product="18S ribosomal RNA"
primer bind 1..20
ORIGIN
1 aacctggttg atcctgccag tagtcatacg ctcgtctcaa agattaagcc atgcatgtct
61 aagtataaat ctcttacttt gaaactgcga acggctcatt atatcagtta tagtttattt

Figure 9C: shows how to save the GenBank full record file

Step5: Save all GenBank (gbk) files in “ta-output/gbkFiles” subdirectory/subfolder and run “ta.pl” as shown in figure 10. With choice 3, this program parses the GenBank file(s) and list organism information in file “taxonomictable.txt” under “ta-output/gbkParsingOutput” subdirectory/subfolder. Apparently ta.pl program assigns the taxonomic group/path to the newly sequenced 454 sequences. The final output of the ta.pl program is found in “ta-output/ taxonomicAssignmentOutput” subdirectory/subfolder.

```
i122mc100:CANGS1.1 ramvinay$ perl ta.pl CANGSOptions.txt

        ****** TAXONOMIC ANALYSIS PROGRAM MENU ******

        1. BLAST SEARCH [1]

        2. BLAST OUTPUT PARSING [2]

        3. PARSING GEN BANK FILES & ASSIGNING THE TAXONOMIC PATH TO 454 SEQUENCES [3]

        4. Press [RETURN] to exit the program

        Your Choice: 3
```

Figure 10: shows how to run the program for GenBank file parsing & creating final tabular output

6.2 ba.pl -- (Blast Analysis) program: This program produces sequence frequency table, which gives an idea of how much overlap there is in species composition between different samples, which will give an idea of the species turnover and fluctuations in diversity.

This program works in following way:

1) Creates BLAST database by *formatdb* program of standalone BLAST (option: "BLAST_DIRECTORY").
2) Runs *blastn* program of standalone BLAST for each sequence against all sequences of all samples.
3) Parses blast search output (option: "BLAST_SIMILARITY_CUTOFF").
4) Creates frequency table for each sample.
5) Trims forward and reverse primer sequences

Parsing Criteria:

1) Gaps are not considered to calculate the percent similarity.

Thus the formula for calculating the percent similarity is:

$$\% \textit{ Similarity} = \textit{Total alignment length} / \textit{Query length}$$

2) The frequency between 2 sequences is calculated by taking the lower number of overlapping sequences.

Example: If query sequence “QuerySample1seq10count120” gets hit with “TargetSample1seq1count13” then 13 will be taken as frequency between these 2 sequences.

Blast Analysis program (ba.pl) Output:

Query Sample Sequence	Query Sample	Target Sample 1	Target Sample 2	Target Sample 3	Target Sample 4	Target Sample 5
seq2count4729	4729	579	665	130	91	221
seq3count2728	2728	77	28	166	275	470
seq4count1298	1298	227	209	137	572	196
seq6count698	698	91	120	40	82	2
seq7count579	579	1	8	132	23	5
seq8count510	510	510	510	510	281	32
seq9count504	504	363	0	0	0	0
seq10count496	496	496	496	198	221	14
seq11count486	486	291	81	51	68	16
seq12count478	478	114	1	0	0	0
seq14count364	364	364	26	0	1	0
seq15count331	331	331	99	7	28	9
seq16count317	317	317	111	273	317	82
seq17count310	310	310	141	138	5	21
seq19count263	263	263	205	263	89	42
seq20count226	226	226	226	226	226	226
seq21count215	215	215	51	7	8	8
seq22count172	172	0	0	0	0	0
seq23count163	163	163	27	0	163	121
seq24count148	148	134	81	108	148	39
seq25count146	146	75	45	17	146	117
seq26count136	136	136	136	86	136	99

Figure 11: shows the partial output of the ba.pl program

How to run the Blast Analysis (ba.pl) program?

Customize the following input options for ba.pl program in “*CANGSOptions.txt”* file and follow the given steps below:

```
##########################################################################################
## ba.pl options
##########################################################################################

# ba.pl input sequences sub directory.
BLAST_INPUT_DIRECTORY    ba-input

# ba.pl output sub directory
BLAST_OUTPUT_DIRECTORY  ba-output

# The executable BLAST location
# Enter the full path to the directory containing the BLAST executables
BLAST_DIRECTORY blast/bin

# The cutoff BLAST Similarity to find the fluctuation and turnover
# of the species in different samples.
BLAST_SIMILARITY_CUTOFF 99
```

Figure 12: shows the input options for blast analysis (ba.pl) program

Step1: Put all non-redundant FASTA sequence files in “ba-input” subdirectory/subfolder.

Step2: Run the Program “ba.pl” as shown below in figure 13, if you mentioned the BLAST similarity cutoff value (For Example: 100,99,98 etc.) in “*CANGSOptions.txt”* file, ELSE go to Step 3.

```
i122mc100:CANGS1.1 ramvinay$ perl ba.pl CANGSOptions.txt 99

        ****** BLAST ANALYSIS PROGRAM MENU ******

        1. TO GROUP SEQUENCES EXACTLY WITH 99% SIMILARITY [1]

        2. TO GROUP SEQUENCES FROM 99% TO 100% SIMILARITY [2]

        3. Press [RETURN] to exit the program

        Your Choice: 1
```

Figure 13: shows how to run the Program to get the Blast Analysis Output (by giving % similarity cutoff value in input file)

Step3: Run the Program "ba.pl" as shown below in figure 14. In this, give the % similarity cutoff value on command line (For Example: 100, 99, 98 etc.). The final output of the ba.pl program is found in "ba-output/blastSearchOutput" subdirectory/subfolder.

```
i122mc100:CANGS1.1 ramvinay$ perl ba.pl CANGSOptions.txt 99

        ****** BLAST ANALYSIS PROGRAM MENU ******

        1. TO GROUP SEQUENCES EXACTLY WITH 99% SIMILARITY [1]

        2. TO GROUP SEQUENCES FROM 99% TO 100% SIMILARITY [2]

        3. Press [RETURN] to exit the program

        Your Choice: 2
```

Figure 14: shows how to run the Program to get the Blast Analysis Output (by giving % similarity cutoff value on command line)

6.3 ra.pl -- (Rarefaction Analysis) program: This program returns the results of rarefaction analysis performed by two freely available software packages (Mothur 1.3.0 and Analytic Rarefaction), which have been integrated into the CANGS procedure.

How does the ra.pl program integrate MOTHUR?

1) Creates non-redundant sequence library.
2) Calculates pair wise distance, using MAFFT program (option: "MAFFT_EXECUTABLE").
3) Creates full square matrix by expanding the unique sequence in actual sequence.
4) Executes the MOTHUR program.
5) Creates output.

How does ra.pl program generate input for Analytic Rarefaction?

1) Creates non-redundant sequence library.
2) Calculates the abundance of non-redundant sequences.

How to run Analytic Rarefaction with the input generated by ra.pl?

3) Rename the input file as rarefaction.dat.
4) Run the Analytic Rarefaction program by double click.
5) Analytic Rarefaction creates output.

numsequences	unique	lci	hci	0.01	lci	hci
1	1.00	1.00	1.00	1.00	1.00	1.00
100	44.08	36.08	52.07	43.25	35.44	51.05
200	68.00	57.61	78.40	66.36	56.48	76.24
300	86.86	75.07	98.65	84.24	73.17	95.32
400	103.03	90.70	115.35	99.11	86.73	111.50
500	117.12	104.12	130.13	112.25	98.81	125.70
600	130.14	116.30	143.98	124.14	110.05	138.24
700	142.32	127.65	156.98	135.55	120.69	150.40
800	153.98	138.61	169.35	146.11	130.72	161.50

A

n	E	Var	Upper95	Lower95	Upper99	Lower99
5000	638.5	283.27	671.46	605.48	681.89	595.05
10000	954.6	417.41	994.67	914.59	1007.34	901.92
15000	1204.6	510.46	1248.92	1160.35	1262.93	1146.34
20000	1417.8	577.86	1464.94	1370.71	1479.84	1355.80
25000	1605.9	626.83	1654.99	1556.85	1670.51	1541.32
30000	1775.1	661.67	1825.54	1724.70	1841.49	1708.76
35000	1929.3	685.27	1980.56	1877.94	1996.79	1861.71
40000	2070.9	699.72	2122.73	2019.03	2139.13	2002.63
45000	2201.9	706.61	2253.95	2149.75	2270.44	2133.27

B

Figure 15: shows A) partial output of the MOTHUR program and B) partial output of the Analytic Rarefaction program

How to run the Rarefaction Analysis (ra.pl) program?

Customize the following input options for ra.pl program in "*CANGSOptions.txt"* file and follow the given steps below:

```
##########################################################################################
## ra.pl options
##########################################################################################

# ra.pl input sequences sub directory
RA_INPUT_DIRECTORY  ra-input

# ra.pl output sub directory
RA_OUTPUT_DIRECTORY ra-output

# The location of MAFFT executable program
# Enter the full path to the directory containing the MAFFT executables (mafft-distance)
MAFFT_EXECUTABLE    mafft-6.716/binaries

# The location of MOTHUR executable program
# Enter the full path to the directory containing the MOTHUR executables.
MOTHUR_EXECUTABLE   mothur

# If PAIRWISE_DISTANCE_CUTOFF is 0.03 that means MOTHUR will estimates OTUs (species)
# sequences with 0%, 1%, 2% and 3% difference.
PAIRWISE_DISTANCE_CUTOFF  0.03

# There options are there 1) furthest 2) nearest and 3) average
SEQUENCE_CLUSTERING_METHOD  furthest

# IF FREQUENCY_TO_OUTPUT_OTUs is given as 1000 that means you only want to output
# the data every 1,000 sequences
FREQUENCY_TO_OUTPUT_OTUs    100

# The executable BLAST location
# Enter the full path to the directory containing the BLAST executables
BLAST_DIRECTORY blast/bin
```

Figure 16: shows the input options for rarefaction analysis (ra.pl) program

Running MOTHUR program:

Step1: Put all non-redundant FASTA sequence files in “ra-input” subdirectory/subfolder.

Step2: Run the rarefaction analysis program (ra.pl) as shown below in figure 17, if you have kept the input sequence fasta file(s) in “ra-input” subdirectory, then go to step 3. With choice 1 this program will run the MOTHUR analysis pipeline.

For Example: > perl ra.pl CANGSOptions.txt

```
i122mc100:CANGS1.1 ramvinay$ perl ra.pl CANGSOptions.txt

            ****** RAREFACTION ANALYSIS PROGRAMS MENU ******

            1. MOTHUR pipeline [1]

            2. Analytic Rarefaction pipeline [2]

            3. Press [RETURN] to exit the program

            Your Choice: 1
```

Figure 17: shows how to run the MOTHUR Analysis Program (by putting sequence files in ra-input subdirectory)

Step3: Run the rarefaction analysis program (ra.pl) as shown below in figure 18. It may be better to give as input options. In this, give the sequence file with full path on command line. With choice 1 this program will run the MOTHUR analysis pipeline. The final output of the ra.pl program for MOTHUR analysis pipeline is found in "ra-output/outputMothur" subdirectory/subfolder.

For Example: > perl ra.pl CANGSOptions.txt ra-input/allsequences.fasta

```
i122mc100:CANGS1.1 ramvinay$ perl ra.pl CANGSOptions.txt ra-input/allsequences.fasta

        ****** RAREFACTION ANALYSIS PROGRAMS MENU ******

        1. MOTHUR pipeline [1]

        2. Analytic Rarefaction pipeline [2]

        3. Press [RETURN] to exit the program

        Your Choice: 1
```

Figure 18: shows how to run the MOTHUR Analysis Program (by giving sequence file on command line)

Generating Analytic Rarefaction input:

Step1: Put all non-redundant FASTA sequence files in "ra-input" subdirectory/subfolder.

Step2: Run the rarefaction analysis program (ra.pl) as shown below in figure 19 if you have kept the input sequence fasta file(s) in "ra-input" subdirectory ELSE go to Step 3. With choice 2 this program will run the <u>Analytic Rarefaction analysis pipeline</u>.

For Example: > perl ra.pl CANGSOptions.txt

```
i122mc100:CANGS1.1 ramvinay$ perl ra.pl CANGSOptions.txt

        ****** RAREFACTION ANALYSIS PROGRAMS MENU ******

        1. MOTHUR pipeline [1]

        2. Analytic Rarefaction pipeline [2]

        3. Press [RETURN] to exit the program

        Your Choice: 2
```

Figure 19: shows how to create the input for Analytic Rarefaction Program (by putting sequence files in "ra-input" subdirectory)

Step3: Run the rarefaction analysis program (ra.pl) as shown below in Figure 20. In this, give the sequence file with full path on command line. With choice 2 this program will run the <u>Analytic Rarefaction analysis pipeline</u>. The final input of the Analytic rarefaction program is found in "ra-output/outputAnalyticRarefaction" subdirectory/subfolder.

For Example: > perl ra.pl CANGSOptions.txt ra-input/allsequences.fasta

```
i122mc100:CANGS1.1 ramvinay$ perl ra.pl CANGSOptions.txt ra-input/allsequences.fasta

        ****** RAREFACTION ANALYSIS PROGRAMS MENU ******

        1. MOTHUR pipeline [1]

        2. Analytic Rarefaction pipeline [2]

        3. Press [RETURN] to exit the program

        Your Choice: 2
```

Figure 20: shows how to create the input for Analytic Rarefaction Program (by giving sequence file on command line)

Step4: Rename the output file, which is created in step2 or Step3 as “rarefaction.dat”. Put this file in the same location where the Analytic Rarefaction program is located and run the “Analytic Rarefaction” program by double click.
